# Profiling chromatin regulatory landscape: insights into the development of ChIP-seq and ATAC-seq

**DOI:** 10.1186/s43556-020-00009-w

**Published:** 2020-10-10

**Authors:** Shaoqian Ma, Yongyou Zhang

**Affiliations:** School of Life Sciences, Xiamen University, Xiamen, 361102 Fujian China

**Keywords:** Chromatin regulatory landscape, Epigenome sequencing, Single cell, Developmental biology

## Abstract

Chromatin regulatory landscape plays a critical role in many disease processes and embryo development. Epigenome sequencing technologies such as chromatin immunoprecipitation sequencing (ChIP-seq) and assay for transposase-accessible chromatin with high-throughput sequencing (ATAC-seq) have enabled us to dissect the pan-genomic regulatory landscape of cells and tissues in both time and space dimensions by detecting specific chromatin state and its corresponding transcription factors. Pioneered by the advancement of chromatin immunoprecipitation-chip (ChIP-chip) technology, abundant epigenome profiling technologies have become available such as ChIP-seq, DNase I hypersensitive site sequencing (DNase-seq), ATAC-seq and so on. The advent of single-cell sequencing has revolutionized the next-generation sequencing, applications in single-cell epigenetics are enriched rapidly. Epigenome sequencing technologies have evolved from low-throughput to high-throughput and from bulk sample to the single-cell scope, which unprecedentedly benefits scientists to interpret life from different angles. In this review, after briefly introducing the background knowledge of epigenome biology, we discuss the development of epigenome sequencing technologies, especially ChIP-seq & ATAC-seq and their current applications in scientific research. Finally, we provide insights into future applications and challenges.

## Introduction

The genome is packaged by histone proteins that are decorated with a wide variety of modifications. Histone acetylation is one of the best-characterized chromatin modifications and correlates with the opening of local chromatin structures and transcriptional activation (e.g., H3K27ac correlates with enhancers) [1]. Compared with histone acetylation, histone methylation is more diverse in terms of both functions and forms. Histon methylation includes H3K4me1, H3K4me3, H3K9me3, H3K36, H3K79, etc. Specific methylation of lysines can exist as monomethylation, dimethylation or trimethylation with different functions [2]. Repressive histone methylation, such as H3K9me3, is highly associated with condensed and constitutive heterochromatin [3]. Meanwhile, active histone methylation such as H3K4me3 contributes to active transcription. Several studies even revealed a class of bivalent chromatin with both active and repressive features, which exhibits overlapping patterns of H3K4me3 and H3K27me3 [4]. The discovery of the bivalent signature of such poised genes was unexpected and very important. For instance, it can be a critical landmark in the maternal-to-zygotic transition process, providing the first clues about the “intermediate” state [5]. Also, bivalent chromatin is not specific to embryonic stem cells and has been well documented in other cell types [6, 7]. In more complex cases, elements that behave as promoters in some tissues can act as enhancers in other tissues, called cREDS (cis-regulatory elements with dynamic signatures), and the same regulatory elements can have both promoter and enhancer signatures [8].

More histone modifications also include phosphorylation, ubiquitination, sumoylation and ADP ribosylation. The current exploration of histone modifications still has the following problems:

1. Modifications on histone surfaces are often dynamic: some modifications can be added and erased in just a few minutes after a cell has been stimulated, so that the histone modifications detected in a population of cells under specific conditions at a given moment are in fact only partially representative of the types of potential modifications.
2. Antibodies to detect histone modifications are essential for many epigenetic chromatin sequencing techniques: in ChIP-seq, for example, antibody specificity testing is required for both histones and transcription factors [9].
3. The mechanisms of heterochromatin formation and diffusion, and the “memory” and “fading” of histone modifications, have been studied only to a minimal extent.
4. The concept of histone code can not be widely used to accurately describe and predict a specific biological phenomenon in many cases [10, 11].
5. Some histone modifications are active in some genomic regions. In contrast, repressive in others: for example, methylation of the H3K9 locus can be repressive in the promoter region and active in the coding region [12].

In contrast to single histone modifications, the chromatin regulatory landscape is a higher-level annotation on the biological function of chromatin that combines histone modifications, transcription factor binding, and the regulatory function of genomic elements. The renowned Roadmap Epigenomics Consortium has led large-scale human reference epigenomic studies that have provided detailed and accurate descriptions and classifications of the functional states of regulatory elements [7, 13]. In addition, by combining these chromatin epigenomic states with existing genome-wide methylation information and gene expression profiles from RNA sequencing (RNA-seq) [14], scientists can interpret the tissue-specific epigenomic landscape from a multi-dimensional perspective.

Many studies of chromatin dynamics require, besides access to information on histone modifications and genomic regulatory elements, a proper understanding of the interactions between transcription factors and chromatin, which is often essential for understanding development and disease progression [15, 16]. Some transcription factors binding to chromatin regulatory regions require specific histone modifications, while others require the assistance of open chromatin and other activators. Binding of some transcription factors to the regulatory regions facilitates the recruitment of additional transcription factors. It may promote the diffusion of chromatin status, which in turn further influences transcription factor binding. The in-depth research on the interaction mechanism is still limited. Recent research has explored epigenetic regulatory mechanisms by constructing synthetic read-write modules, which will help us better understand the basic principles of epigenetic inheritance [17].

## The development of technologies for investigating chromatin dynamics

### ChIP-based methods

The earliest technology applied to large-scale epigenetic mapping was chromatin immunoprecipitation (ChIP) followed by microarray hybridization (chip) (ChIP-chip), which allowed scientists to detect DNA-protein interactions on a genome-wide scale [18]. ChIP-chip is based on microarray hybridization, where a large number of probes covering a genome or a specified region are seeded on a high-density chip. However, this method has a few shortcomings: low resolution, ambiguous factors introduced by the probe design, signal bias, difficult in broad application to more species [19].

Compared to Chip-chip, chromatin immunoprecipitation sequencing (ChIP-seq) provides higher resolution, less noise, and greater coverage [20, 21]. With the rapid decline in the cost of second-generation sequencing, ChIP-seq will become one of the indispensable tools for studying gene regulation and epigenetics. In addition to better identification of sequence motifs, ChIP-seq can also be utilized to find key transcription factors, enhancers, and other regulatory elements [22].

#### Conventional ChIP-seq

**Fig. 1:**
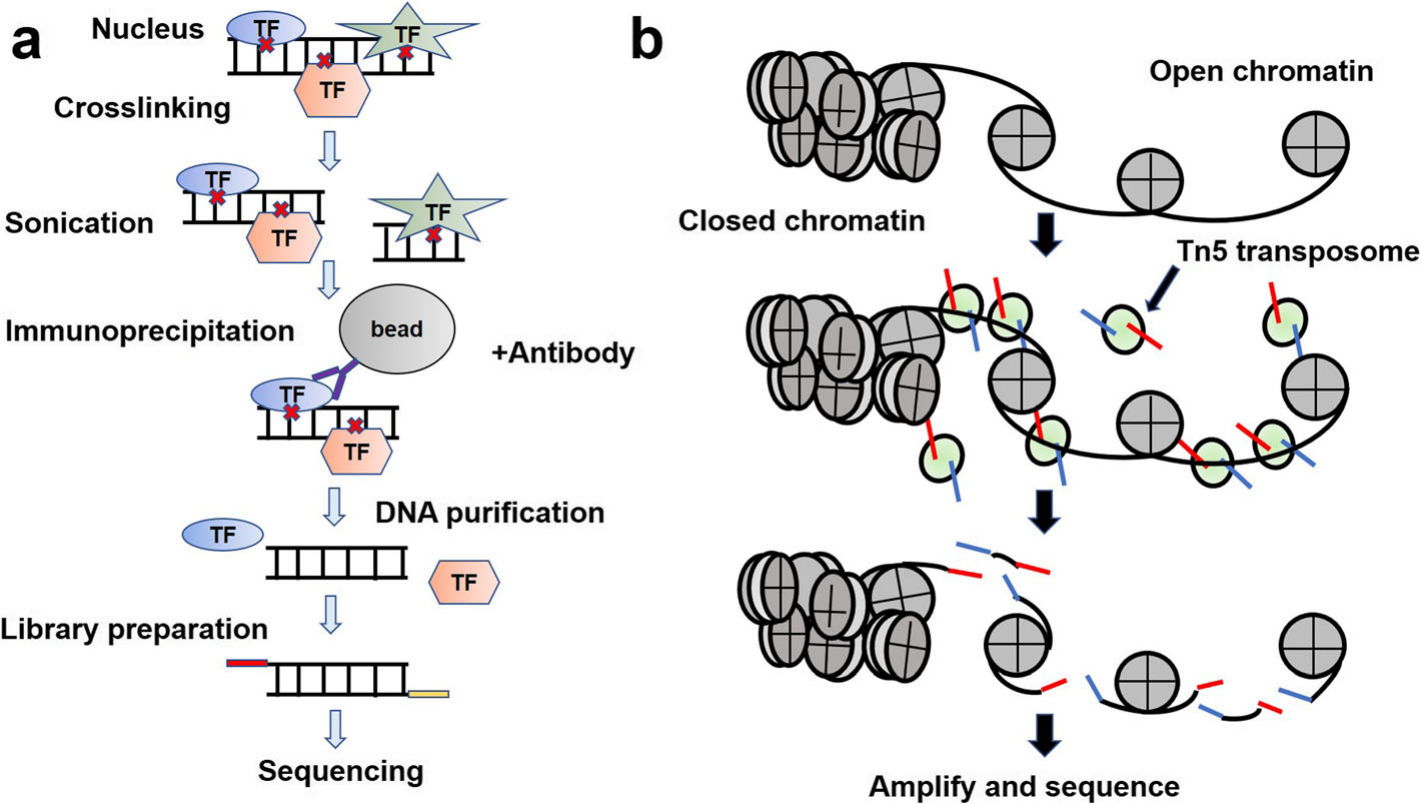
Workflows of ChIP-seq and ATAC-seq. **a** In ChIP-seq, chromatin is crosslinked using formaldehyde and sonicated to obtain DNA fragments of 200–600 base pairs. Then the DNA-protein complex of interest can be immunoprecipitated by the antibody. Library preparation steps: end repair, A-tailing and adapters ligation, library sequencing. **b** ATAC-seq identifies regions of open chromatin using a hyperactive Tn5 transposase, which preferentially inserts into accessible chromatin and tags the sites with sequencing adaptors

For DNA-binding proteins, ChIP-seq experiments are aimed at enriching DNAs bound to specific proteins. The procedure consists of multiple steps (Fig. 1a): first crosslinking DNA and proteins in situ via formaldehyde, followed by sonication of the DNA into small 200–600 bp fragments, and then immunoprecipitation of the DNA-protein complexes of interest with antibodies. The DNA is then uncross-linked, and the released DNA is subjected to end repair, adapter ligation and other library preparation steps. Finally, sequencing is performed.

However, ChIP-seq has some limitations: the introduction of potential bias by PCR amplification, length limitations of PCR amplification, GC bias in both fragmentation and sequencing processes, the need for 10^5^ ∼ 10^7^ cells due to a massive loss in the immunoprecipitation process, and the potential for epitope masking due to the formaldehyde crosslinking process [23]. Formaldehyde can crosslink transcription factors to DNA, maintaining their binding status in vivo, but it may also mask the antigenic epitopes of the transcription factors, producing false-positive results. This formaldehyde crosslinked ChIP is also known as X-ChIP [20]. Natural ChIP (N-ChIP), on the other hand, does not have the problem of epitope masking and can be used for a much smaller number of cells [24, 25]. This method abandons formaldehyde fixation, using micrococcal nuclease (MNase) for chromatin digestion. MNase can quickly and gently sever chromatin DNA and maximize the preservation of the original chromatin structure and the binding of the target protein, increasing the reliability of the ChIP results. However, this approach may result in the loss of many binding sites when target proteins do not bind strongly to DNA, so it is generally used for ChIP of histones other than transcription factors [26]. For antibody-dependent epigenetic sequencing techniques, the selection of the appropriate antibody is often the most critical step. In terms of choosing between monoclonal or polyclonal antibodies, monoclonal antibodies may result in weak signals due to interference from other protein components. In contrast, polyclonal antibodies may produce unwanted false positives [23].

#### Lowering cellular input

A major limitation of conventional ChIP-seq is the large number of cells required (10^5^ ∼ 10^7^ cells). Various strategies have been adapted to optimize the protocol to measure low-abundance cells over the past few years. ChIPmentation combines chromatin immunoprecipitation with sequencing library preparation by Tn5 transposase. The fast, low-cost library preparation protocol allows histone ChIP-seq using 10,000 cells [27]. The ultra-low-input MNase-based native ChIP (ULI-NChIP) generates high-quality maps of histone modifications from 10^3^ to 10^6^ embryonic stem cells by adjusting NChIP procedure [28]. Microfluidic oscillatory washing-based ChIP-seq (MOWChIP-seq) can even be applied to genome-wide analysis of histone modifications using as few as 100 cells [29]. While these methods can yield relatively accurate profiles of histone modifications such as H3K4me3 in a small number of cells, they are ineffective for many transcription factors with significantly fewer binding sites across the genome.

Cleavage under targets and release using nuclease (CUT&RUN), like ChIP-seq, is for detecting DNA-protein interactions, and it does not require formaldehyde crosslinking and sonication-based fragmentation, but instead uses MNase fused to Protein A/G to cut and release target DNA fragments in situ, thus significantly increasing the signal-to-noise ratio, and can be applied to as low as 100 ~ 1000 cells [30]. The ultra-low-input CUT&RUN (uliCUT&RUN) further improves this method to the single-cell level. By using this method, researchers found that the binding sites of CTCF in hESC cells were more concentrated, while H3K4me3 occupied a relatively wide region. Also, the binding pattern of SOX2 and Nanog, as well as other essential transcription factors in early embryonic development, were precisely described [31].

Cleavage under targets and tagmentation (CUT&Tag) is another technology that can detect DNA-protein interaction in low cellular input samples or even single cells [32]. Instead of Protein A/G-fused MNase, CUT&Tag applies Protein A/G-fused Tn5 transposase (pA/G-Tn5) to cut DNA, demanding high-quality core enzyme. The core enzyme pA/G-Tn5 has high activity, high sensitivity and high affinity for trace amounts of DNA and can effectively capture limited binding sites in a small number of cells. Another great feature of CUT&Tag is that all the library preparation steps are performed in the same tube after the addition of concanavalin protein A beads. As a result, the sequencing data has a lower background. So compared to CUT&RUN, CUT&Tag without end repair and additional adapter ligation is easier and more efficient. Since pA/G-Tn5 can bind and cut non-specific open chromatin under different conditions, CUT&Tag has the potential to simultaneously access some of the open chromatin and specific transcription factor binding sites by changing buffers composition. However, non-specific DNA cutting is a disturbance to the desired transcription factor binding profile in more scientific contexts, where the known open chromatin data need to be combined to remove these disturbances. Too complicated data cleaning process is generally not recommended, as combining other data to remove background often risks introducing batch effects.

#### Single-cell ChIP-seq

**Fig. 2:**
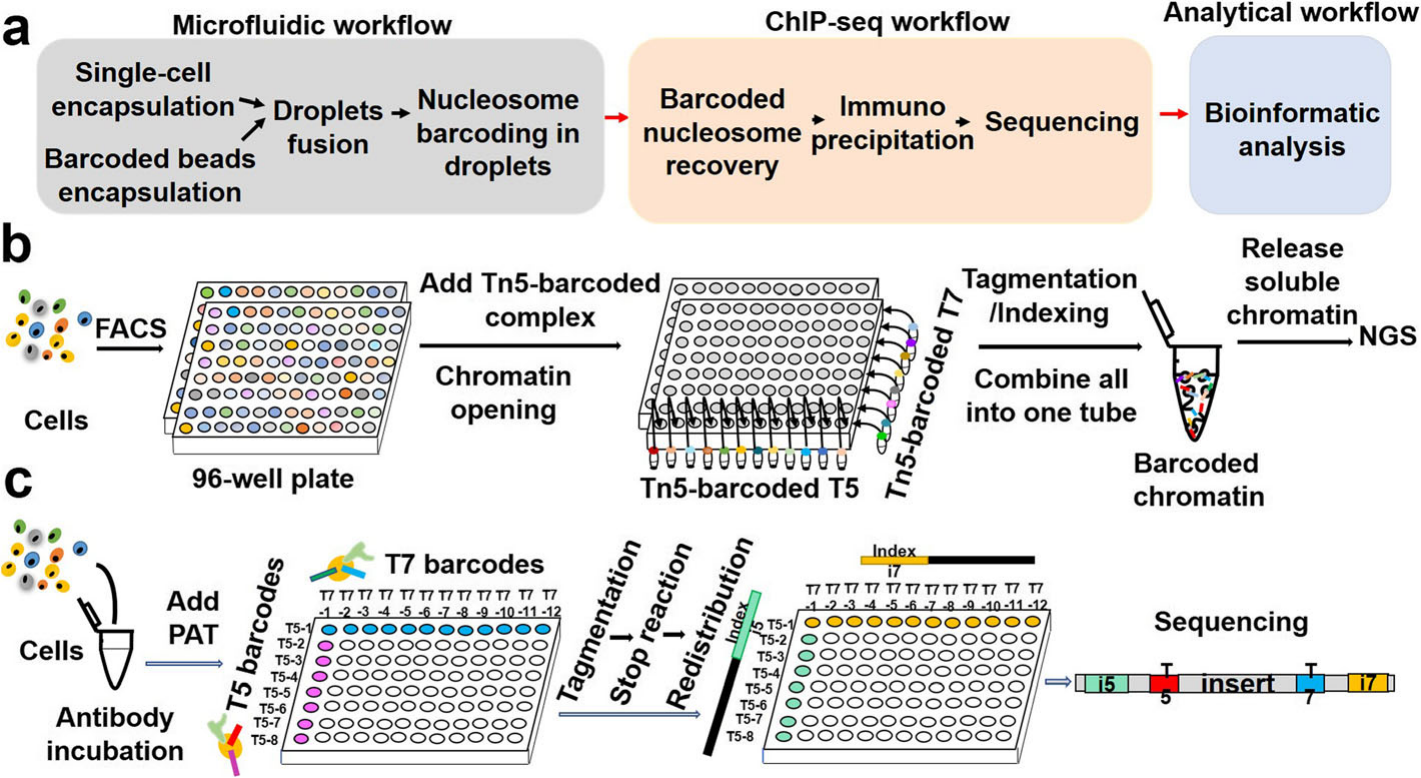
Workflows of single-cell ChIP-seq. **a** Workflow of Drop-ChIP. **b** Workflow of sc-itChIP-seq. FACS: fluorescence-activated cell sorting. NGS: next-generation sequencing. **c** Workflow of coBatch. PAT: the fusion of the N-terminal of Tn5 transposase with protein A (pA-Tn5 [PAT])

In contrast to bulk ChIP-seq, which is unable to detect chromatin signatures of individual cells, single-cell ChIP-seq (scChIP-seq) helps study genetic diversity in heterogeneous cell populations and understand the evolution of tumor populations. Droplet-based single-cell ChIP-seq (Drop-ChIP) combines a microfluidic device with single-cell DNA barcoding, allowing researchers to obtain a relatively low-coverage map per cell [33]. The basic experimental procedure consists of four steps (Fig. 2a): 1) Droplet formation: each cell encapsulated in a droplet is mixed with lysate and MNase; cells are then lysed in drops and their chromatin is fragmented; the second barcode drop contains DNA barcodes for ligation to the chromatin fragments. The two drops are mixed to form an indexing microreactor. 2) Nucleosome barcoding in droplets. 3) Immunoprecipitation of barcoded nucleosomes. 4) Library construction and sequencing. The downstream data analysis pipeline is similar to that of single-cell RNA-seq [34]. scChIP-seq enables the clustering of cell populations based on the diversity of the chromatin landscape and the identification of chromatin features specific to each population, such as the loss of H3K27me3 markers in some cells may associate with chemoresistance [35]. However, because the data generated by Drop-ChIP of individual cells are too sparse, thousands of cells are required to obtain good results for clustering.

Epigenomic tools that largely reduce cellular input, including CUT&RUN and CUT&Tag, do not necessarily perform better than ChIP-seq, depending on the situation. Both single-cell CUT&Tag and single-cell ATAC-seq (scATAC-seq) rely on a special device called Takara ICELL8 [36]. The microfluidic-based Drop-ChIP can only generate about 1000 unique reads per cell, the too sparse data coupled with expensive devices limit its application [33]. Therefore, ChIP methods that can be applied more widely, perform more robustly, and cost less on low-abundance cells or even a single cell are urgently needed. Single-cell simultaneous indexing and tagmentation-based ChIP-seq (sc-itChIP-seq) is a ChIP-based technology with high-resolution and its experimental procedure begins with a whole-genome chromatin opening of fixed cells/native cells sorted by FACS (fluorescence activated cell sorting), allowing Tn5 to fragment DNA homogeneously [37]. The released DNA fragments are enriched by immunoprecipitation (Fig. 2b). DNA-protein interaction peaks of individual cells can be obtained by demultiplexing and debarcoding. The library preparation steps are all carried out in the same tube, which dramatically reduces the loss. sc-itChIP-seq is useful for the identification of lineage-specific enhancers and key transcription factors during the differentiation process. Scientists have applied this approach to successfully identify the epigenetic trajectory of naive mouse embryonic stem cells and to reveal the reprogramming events of the lineage-specific enhancers in the fate determination of cardiac progenitor cells [37]. Given that sc-itChIP-seq does not depend on costly equipment, its application in laboratories is more widespread than the previous scChIP-seq (Table 1).

**Table 1:** Comparisons between single-cell ChIP-seq methods

Methods	Strategy	Device for cell sorting	Cell state	Mapping rate
scDrop-ChIP	ChIP and microfluidic system for droplet-forming	Costly microfluidic device	Native	70% [33]
sc-itChIP-seq	ChIP and Tn5-barcoding (single round)	FACS	Native, fixed	94% [37]
CUT&Tag	ChIP-free, Tn5-barcoding (for single round)	Costly Takara ICELL8	Native	97% [32]
CoBATCH	ChIP-free, Tn5-barcoding (for two rounds)	FACS	Native, fixed	94% [38]

Combinatorial barcoding and targeted chromatin release (CoBatch) can not only profile the epigenetic landscape of samples with relatively low cellular input, but also be applied to the scale of thousands of single cells in the native or fixed state with a high signal-to-noise ratio [38]. This approach applies an enzyme called PAT, which is the fusion of the N-terminal of Tn5 transposase with protein A. Cells incubated with antibodies are distributed into wells (200 ~ 2000 cells per well), PAT with different barcodes are then added for the first round of indexing. All cells are pooled and redistributed to different wells (20 ~ 25 cells per well), and finally amplified with varying primers of PCR for the second round of indexing (Fig. 2c). Using the single-cell data generated from coBatch, researchers successfully achieved high-throughput identification of cell types such as endothelial cells and mesenchymal cells, revealing the heterogeneity of the endothelial cell population in depth. However, the shortcoming of this method is that it cannot be directly applied to samples with input as low as a dozen cells, such as preimplantation embryos [38].

### Nucleosome positioning and chromatin accessibility

One of the most classical technologies for investigating open chromatin regions is DNase I hypersensitive site sequencing (DNase-seq) [39]. DNaseI has endonuclease activity and can be utilized to obtain open chromatin fragments of appropriate length by controlling the cutting efficiency. DNaseI is used to cut DNase-sensitive sites on the genome. Then the digested fragments are amplified, the sequencing data are analyzed for peaks to acquire information on relatively open chromatin regions as well as protein-protected regions, which are usually the sites of transcription factor binding.

MNase-seq is employed to probe nucleosome positioning, and experiments are performed using MNase digestion to fragment chromatin without crosslinking [40]. Unlike DNase, MNase has both exonuclease and endonuclease activity, after binding to open chromatin. It digests DNA right up until it encounters obstacles such as transcription factors and nucleosomes and removes the linker DNA, which make MNase-seq ideal for exploring nucleosome positioning. MNase-seq requires fathoming the appropriate enzymatic conditions, inevitably resulting in difficult to control confounding factors, such as sequence binding preferences and enzymatic activity of the enzyme itself [41]. ChIP-seq, DNase-seq and MNase-seq measure transcription factor mapping, chromatin accessibility and nucleosome positioning, respectively. They all require large amounts of input material and yield ‘averaged’ profiles that are insensitive to cellular heterogeneity, which significantly limits the application of these technologies in some rare and precious samples like early embryos. Moreover, these technologies involve complicated and time-consuming sample preparation and library construction, and cannot directly investigate the interactions between nucleosome positioning, chromatin accessibility, and transcription factor mapping. Specifically, the most considerable limitations are:

1. High cellular input masks heterogeneity between cell populations; the requirement for input material limits the application of DNase-seq and MNase-seq in specific clinical samples and makes them difficult to achieve personalized epigenomics studies.
2. To obtain the required amount of cells, cells often undergo an in vitro culture for amplification. However, in vitro culture does not fully mimic in vivo conditions and may further add extraneous factors that may cause alterations in chromatin state, thus increasing the risk of experimental failure.

#### The development of ATAC-seq

Assay for transposase-accessible chromatin with high-throughput sequencing (ATAC-seq) technology successfully achieves simultaneous identification of open chromatin regions, nucleosome positioning, and regulatory motifs while reducing input material to 500 ~ 5000 cells [42]. Using motif inference, scientists have successfully inferred the binding sites of DNA-binding proteins in the B cell line. Also, ATAC-seq has a relatively simple and efficient ‘two-step’ library-preparation procedure: transposition and PCR (Fig. 1b). In ATAC-seq experiments, the nuclei of cells are collected after nucleus-cytoplasm separation, and the chromatin inside the nucleus is fragmented by Tn5 transposase and ligated to sequencing adapters, which largely simplifies the protocol. Tightly packed chromatin DNA is not accessible for transposome, whereas chromatin DNA in open regions is randomly inserted and fragmented by transposome. Fragmented DNAs are collected for subsequent analysis. The most significant innovation of ATAC-seq is based on the application of Tn5 transposase: wide-type Tn5 transposase has low activity in transposition [43, 44]. For better usage in scientific research, after directed evolution, it can become hyperactive Tn5 transposase with increased affinity for DNA. Tn5 transposase can be assembled with designed adapters in vitro to form the active transposome complex. Although Tn5 transposase unavoidably brings about bias due to sequence-dependent binding, this transposition bias can be corrected by the development of computational tools [45].

**Fig. 3:**
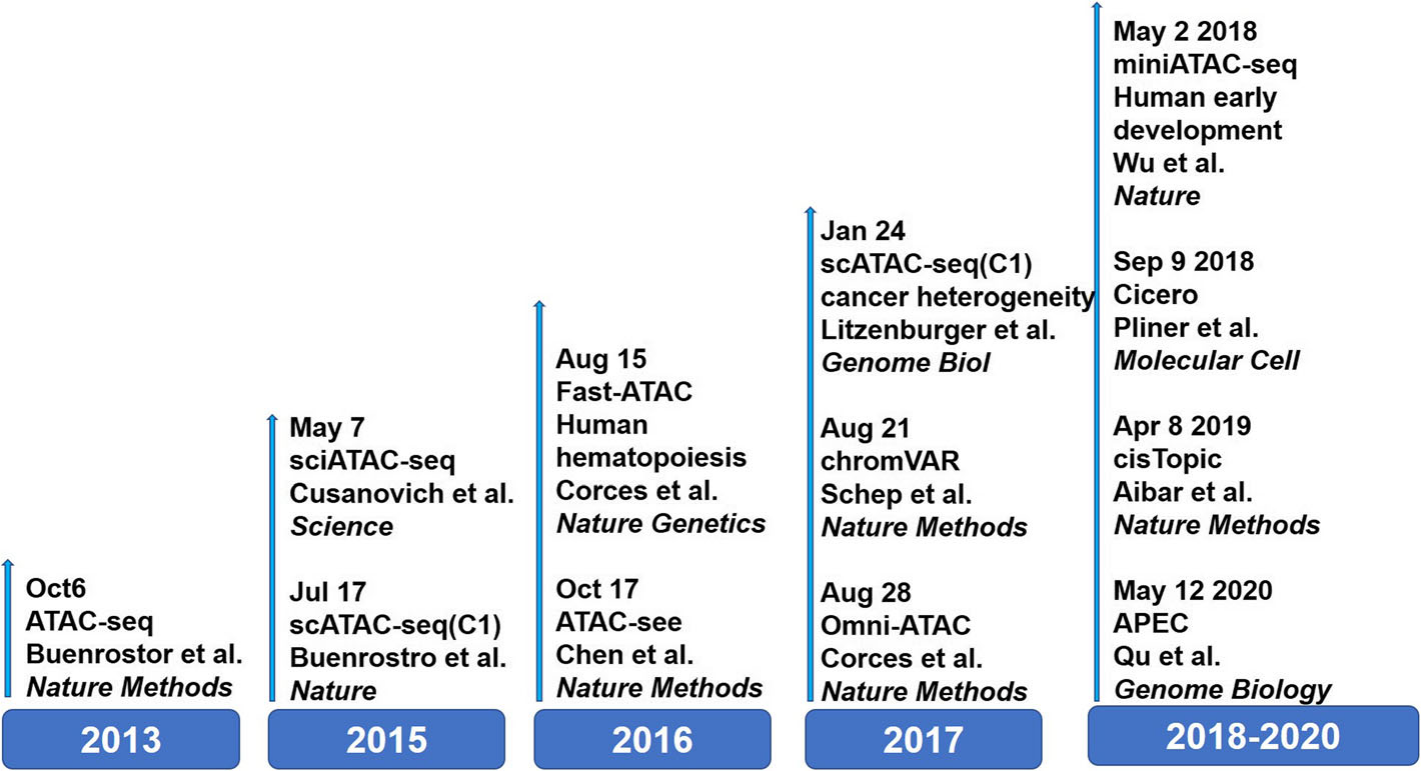
Development of ATAC-seq and analysis tools

ATAC-seq has the advantage of high efficiency and low cellular input requirement, but its applicability to different types of samples is still limited. ATAC-seq-derived technologies generated for expanding applications include fast-ATAC, Omni-ATAC, and miniATAC-seq (Fig. 3) [46–48]. For example, the fast-ATAC is optimized for blood samples, using a digitonin-contained transposition buffer to combine the two steps of permeabilization and transposition into one step. It not only increases the fragment yield per cell but also dramatically reduces the proportion of mitochondrial reads [46]. Omni-ATAC can be applied to a variety of cell types and long-term preserved frozen samples. Improvements, including a variety of different detergents, washing with Tween-20 after cell lysis, and the use of phosphate buffer saline to enhance the signal-to-noise ratio in the transposition reaction. All those improvements allow Omni-ATAC’s application to a wide range of cell lines, tissue types, and frozen samples stored for long periods while improving data quality [47]. miniATAC-seq has been optimized primarily for DNA purification steps and lysis buffer (the optimized concentration of NP-40 for embryos), and can even be applied in 20 cells (e.g. early embryos) with high-quality sequencing data [48]. Optimization of the ATAC-seq protocol has also been extensively used to cells from neural tissues and bio-banked specimens [49, 50].

**Fig. 4:**
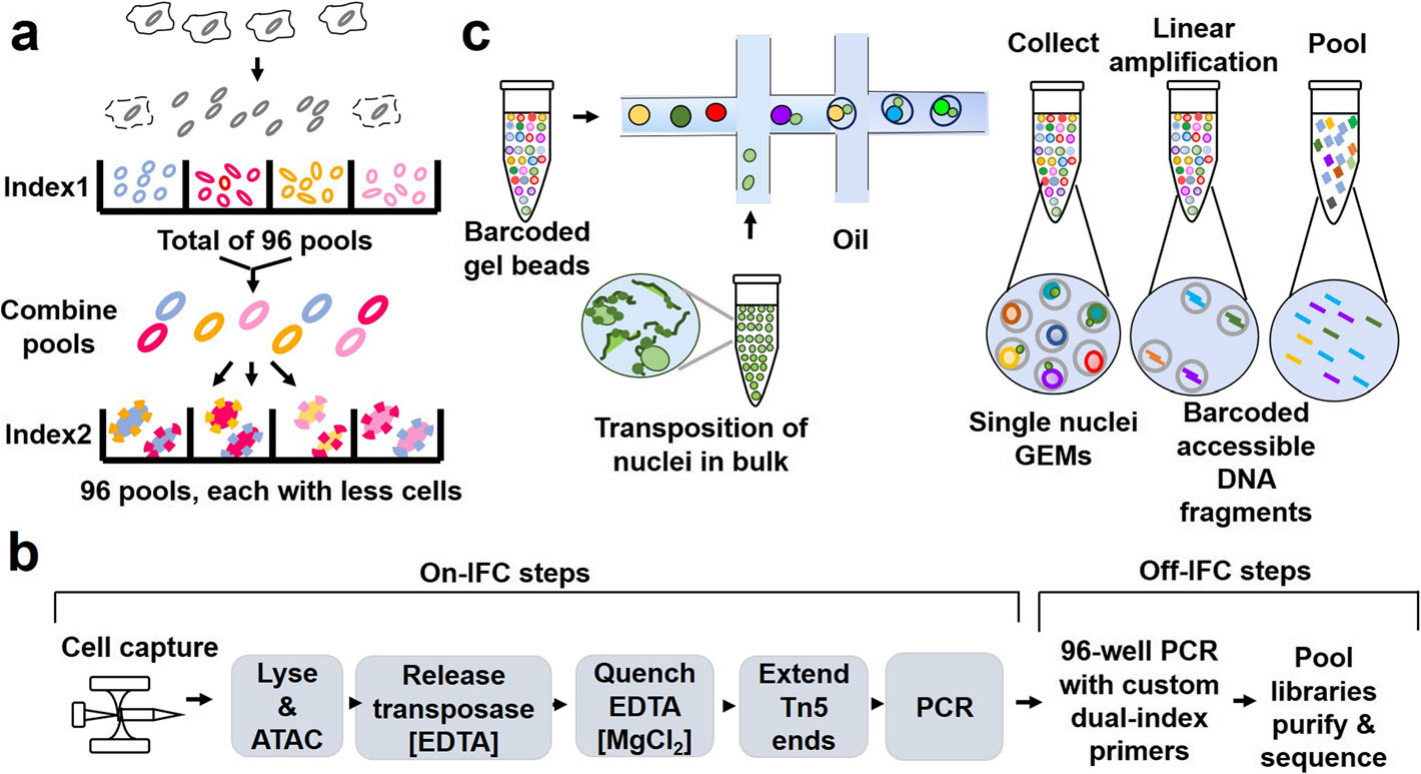
Workflows of single-cell ATAC-seq. **a** The combinatorial indexing method of sci-ATAC-seq. The first barcodes are introduced by Tn5 transposase and the second indexing is introduced by amplification using primers containing a second barcode. **b** scATAC-seq based on the integrated fluidic circuit (IFC). In scATAC-seq using a microfluidics platform (Fluidigm), after transposition and PCR on the IFC, libraries were collected and PCR amplified with cell-identifying barcoded primers. Single-cell libraries were then pooled and sequenced on a high-throughput sequencing instrument. **c** Workflow of droplet-based scATAC-seq (10 × ATAC-seq) implemented on the Chromium platform (10 × Genomics). GEM: gel bead in emulsion

ATAC-seq can measure samples with as low as 500 cells, but it has not been able to decipher cell-to-cell variability at the single-cell level. The advent of single-cell sequencing technology has enabled us to understand life at a finer single-cell level [34, 51]. The information that can be obtained from single-cell transcriptome sequencing is quite limited, and further study of the epigenomic dynamics of regulatory elements at the single-cell level is essential to figure out the more detailed mechanisms of cell differentiation and development. In 2015, the single-cell combinatorial indexing assay for transposase accessible chromatin with sequencing (sci-ATAC-seq) was developed, which enabled the sequencing of a large number of individual cells at the same time [52]. In this strategy, cells were uniquely labeled and sequenced for chromatin accessibility at the single-cell level. After first barcoding all nuclei in the 96-well plate with barcoded Tn5, the nuclei are then pooled and redistributed to a new set of wells. Thus a second barcode could be added by PCR amplification (Fig. 4a). The first scATAC-seq technology based on a microfluidic platform is integrated with Fluidigm single-cell platform C1 (integrated fluidic circuit) (Fig. 4b), it is an automated method for single-cell chromatin accessibility mapping. It can capture more reads per cell than sci-ATAC-seq, opening up the exploring of the diversity of inter-cellular regulators (regulome) [53]. Droplet-based scATAC-seq (10 × ATAC-seq) performed on the Chromium platform (10 × Genomics) achieves a dramatic and unprecedented increase in the throughput of scATAC-seq experiments (Fig. 4c). Single-cell nuclei suspensions are loaded into microfluidics to promote the formation of gel bead in emulsion (GEM). Within each GEM, gel beads oligos were newly designed to consist of a 29-bp sequencing adapter, a 16 bp barcode (to index droplets) and the first 14 bp of read 1 N (primers of the linear amplification reaction), allowing thousands of cells to be measured per experiment. Applying 10 × scATAC-seq, researchers achieved a more excellent mapping of differential open chromatin in immune cells within the tumor microenvironment [54]. To interpret the scATAC-seq data, which are more sparse compared to single-cell RNA-seq data, scientists have developed a variety of computational tools such as chromVAR [55], Cicero [56], cisTopic [57], APEC [58], etc.

#### Interactions between ChIP-seq & ATAC-seq

ChIP-seq alone may be challenging to find meaningful regulatory elements when comparing two or more samples. In this regard, ATAC-seq has the advantage of high resolution and high signal-to-noise ratio, which allows researchers to identify differentially regulated sequences, such as enhancers. Combining ChIP-seq with ATAC-seq makes it easier to determine significant peaks. ChIP-seq methods are still irreplaceable although methods for detecting open chromatin, such as DNase-seq and ATAC-seq can help to infer genomic features. All those techniques are indirect for detecting the binding sites of transcription factors to DNA, which means that the transcription factors mapping inferred from the enriched motifs still needs to be further validated [35, 59]. The probable limitations of using ATAC-seq alone are:

1. Not all chromatin regulators have a corresponding motif, regulators such as chromatin remodeling proteins do not have a DNA sequence preference. In contrast, the interaction between chromatin remodeling proteins and nucleosomes is often an essential factor in early embryonic development as well as cell fate decisions [60]. Open chromatin information alone cannot be used to infer the binding profiles of such factors.
2. Some motifs have the potential to be bound by multiple sequence-specific transcription factors [61].
3. Some of the homologous transcription factors bind in similar motif patterns, in many cases, the direct assignment of open chromatin peaks to specific individual transcription factors is less reliable. Results obtained from direct detection of interactions between specific transcription factors and DNA are often more reliable.

The genome-wide chromatin accessibility profile detected using ATAC-seq represents another level of the chromatin regulatory landscape compared to ChIP-seq, which directly determines specific DNA-protein interactions (Table 2). Therefore, combining ATAC-seq and ChIP-seq will help scientists gain a more comprehensive and in-depth understanding of chromatin regulatory dynamics and their biological significance.

**Table 2:** Comparisons between ChIP-seq and ATAC-seq

Factor	ChIP-seq	ATAC-seq
Dependence on antibody	Yes, ChIP-seq requires good and specific antibodies	No
Throughput for detection	Only for Specific proteins	Global chromatin accessibility
Controls	Required, for distinguishing real peak regions from artifacts	Usually not required, but naked DNA controls are useful for characterizing the sequence bias of enzymatically induced cleavage
Results	Direct interactions between proteins and DNA	Potential binding sites for regulators

## Applications resetting understanding of the biological question

### ChIP-seq & ATAC-seq approaches to refine our understanding of chromatin remodeling in early embryos

**Fig. 5:**
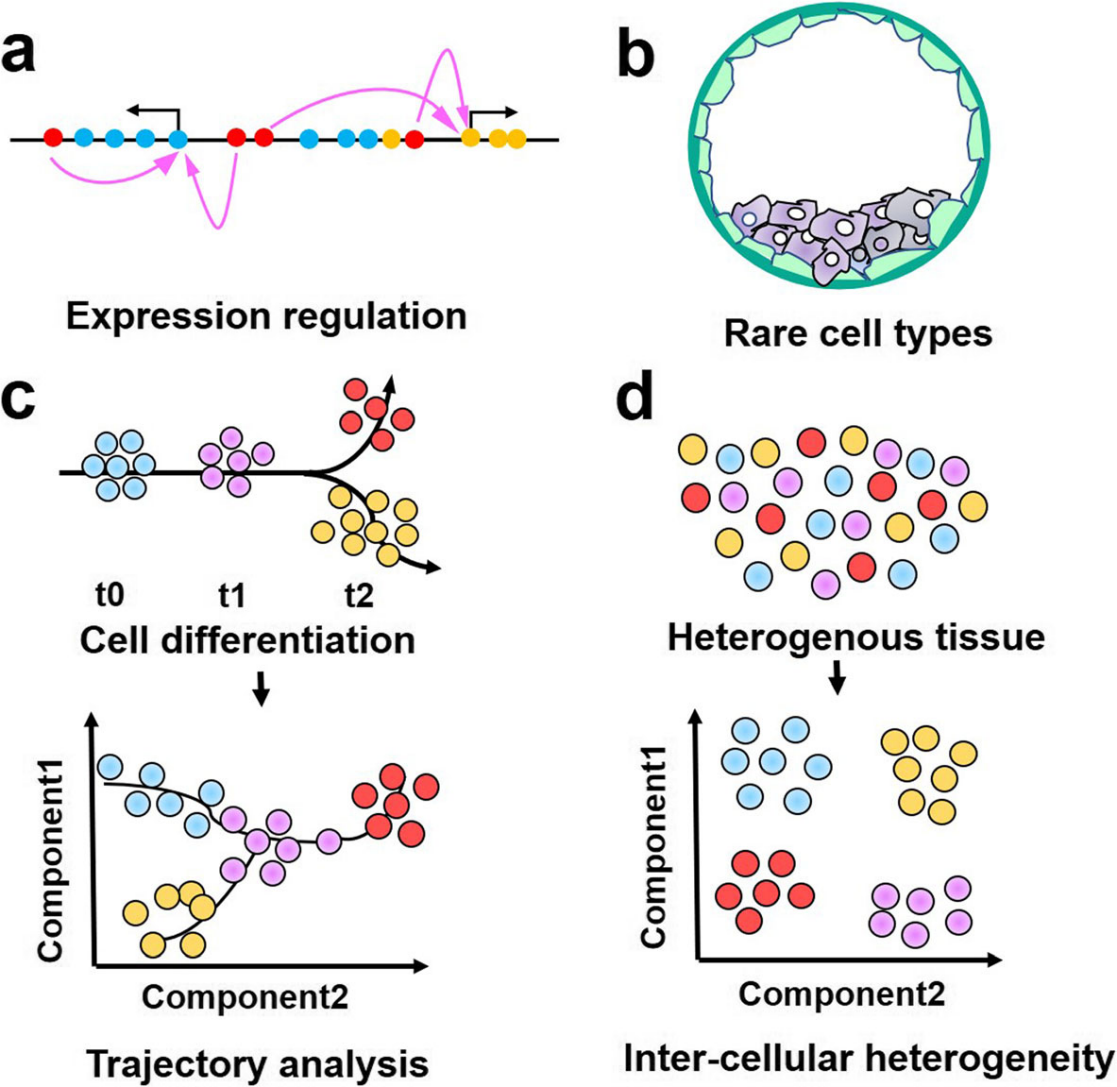
Future applications of single-cell epigenomics. **a** Long-range interaction of regulatory elements. **b** Chromatin dynamics in rare cell types (e.g., early embryos). **c** Cell lineage tracking. **d** Deconvolution of intercellular heterogeneity

Mammalian embryo development involves genome-wide epigenetic changes, including DNA methylation, histone modifications, open chromatin and chromatin conformation [62]. Crucially, many genomic regulatory elements such as promoters, enhancers, insulators, and locus control regions guide embryonic development through interactions with cell type-specific transcription factors [63]. Also, long-range interactions between these regulatory elements are intriguing (Fig. 5a). By innovatively optimizing the protocol of ChIP, it can be available for histone modification reconstruction studies in very low cellular input such as embryos (Fig. 5b). In early mouse embryos, H3K4me3 undergoes extensive reprogramming events, where it disappears in the zygote and is reconstructed again in the offspring, accompanied by zygotic genome activation (ZGA). Thus, the application of ChIP-seq optimization in embryos may help to reveal the detailed process of inheritance of mammalian histone modifications from parents to offspring, i.e., the differences in parental modification patterns before and after fertilization [5, 64, 65]. More importantly, the application of improved ATAC-seq methods in embryonic tissues facilitates the mapping of genome-wide chromatin accessibility at critical periods of embryo development. In preimplantation embryos, for example, ATAC-seq is utilized to reveal the temporal dynamics of high-resolution chromatin changes in ZGA and minor zygotic genome activation (minorZGA) [66]. Additional epigenomic studies have also shown unique chromatin states at different stages of embryo development [67, 68]. These findings provide valuable clues for further interrogating human embryo development and clinical guidance in the future. More questions, such as the key factors and transposons that regulate chromatin state transitions, remain to be discovered.

### Single-cell approaches to probe developmental processes in organs

Epigenome sequencing technology plays an irreplaceable role for experimental sytem to track the developmental trajectories and explore the cell fate determination mechanisms (Fig. 5c). ChIP-seq and ATAC-seq at the single-cell level help to comprehensively resolve the developmental dynamics of tissues and organs. In 2018, scATAC-seq was applied to cluster cells at different stages of mouse forebrain development and used to identify key regulatory factors inferred from the accessible chromatin peaks [69]. Integrating ChIP-seq, ATAC-seq, and DNase-seq in organoid models [70] with transcriptomic data will help to reveal the developmental dynamics of specific cells, discover key transcriptional regulators, and identify disease susceptible cell populations [71–73]. Multi-omics integration of single-cell transcriptome and ATAC-seq in organ development can provide a robust foundation for the clinical treatment of diseases. For example, by revealing the critical time points and gene regulatory networks of human hippocampus development from a comprehensive perspective, researchers provided information on potential cell groups involved in the pathology of Parkinson’s disease, Alzheimer’s disease and Huntington’s disease [74].

In addition to neurobiology studies, chromatin dynamics analysis has shed light on muscle development [75], mammary gland development [76], and cardiac precursor cell fate determination [77]. Fine-grained studies of single-cell chromatin dynamics will help to create a global map of human organ development in the future, enabling us to trace the embryonic origin of every tissue and organ.

### Single-cell approaches to assess the complexity of cancer

There is currently limited understanding of the highly heterogeneous tumor tissues, including differences in the tumor microenvironment, differences between original primary cancer and metastases, and the evolution of tumor subclones. Immune cells in the tumor microenvironment are often critical for the immune escape and infiltration processes of cancer cells [78, 79]. scATAC-seq and its expanded application will help to reveal the heterogeneity of the epigenetic landscape in tumor development and provide potential targets for therapy (Fig. 5d) [80–82]. For example, the application of scATAC-seq has determined the regulatory network of malignant, stromal and immune cells in the tumor microenvironment. The intra-tumor T cell exhaustion in the tumor microenvironment of patients was compared before and after immunotherapy by detecting the dynamics of immune cell development at single cell level [54]. The key regulatory T cell populations that respond to immunotherapy can be identified. Integrated analysis of ChIP-seq, ATAC-seq and DNA mutation profile in the same cell will enable scientists to uncover new subclones of cancer cells, allowing for personalized clinical trials. Thus, understanding the chromatin regulatory landscape at the single-cell level will significantly accelerate biomedical advances in cancer therapy [83].

Extended technologies of ATAC-seq can also provide new insights into tumor heterogeneity. Assay of transposase-accessible chromatin with visualization (ATAC-see) helps to image open chromatin in situ by fluorescently labeling accessible loci [84]. For example, by using ATAC-see and fluorescence in situ hybridization [85], scientists have provided physical evidence of the co-localization of extrachromosomal DNA (ecDNA) [86] and ATAC-see fluorescence signals. Confirmed by ATAC-seq and MNase-seq data, this result suggests that ecDNA is highly accessible, which may explain why oncogenes on ecDNA can be expressed in large quantities [87]. Therefore, the adaptation of ChIP-seq and ATAC-seq technologies can provide new directions for targeted therapies, and supply physical evidence of imaging to the heterogeneity of cancers, making scientific findings more comprehensive and reliable.

## Conclusion and future directions

### Combination of scRNA-seq and scATAC-seq

To construct a complete regulatory network, RNA-seq, ATAC-seq and ChIP-seq often need to be combined. Although many algorithms have been developed to integrate multi-omics data, it is difficult to assess the performance of these algorithms and whether they fully preserve the biological variance. A growing number of studies in recent years have demonstrated the potential of parallel analysis of multiple modalities in the same cells [88–90]. Single-nucleus chromatin accessibility and mRNA expression sequencing (SNARE-seq) based on micro-droplet platform enables simultaneous sequencing of transcriptome and chromatin accessibility in a single cell by dual-omics capturing, and can appropriately correlate the results of both modalities to obtain detailed information on the regulation of gene expression [91]. sci-CAR is a combinatorial indexing–based method that allows co-assay of chromatin accessibility and mRNA (CAR) in thousands of single cells, combining sci-ATAC-seq and sci-RNA-seq [92]. Paired-seq is an ultra-high-throughput method for parallel analysis of transcriptome and accessible chromatin in millions of individual cells [93]. In this method, a ligation-based combinatorial indexing strategy is adopted to simultaneously tag both the open chromatin fragments generated by the Tn5 transposases and the cDNA molecules produced from reverse transcription of RNA in millions of cells. Compared to SNARE-seq and sci-CAR, Paired-seq has a much higher throughput, which makes it possible for analysis of gene regulatory programs at an organismal scale. A significant advantage of open chromatin mapping over the transcriptome [94] is that accessibility maps provide expression status of genes and the network of interactions between regulatory elements, so by combining ATAC-seq with RNA-seq, more compelling biological questions will be solved. For example, spatiotemporal gene expression patterns are highly correlated with *cis*-regulatory elements and thus differ between individual cells. Based on this premise, how these heterogeneous cells act in coordination to construct a comprehensive network of cell communication is intriguing.

### The promise and limitations of probing chromatin in single cells

Compared to open chromatin data derived from populations of cells, scATAC-seq signals are binary and sparse, so a new analytic framework to account for fundamental differences from bulk data is needed. A feasible approach is to aggregate information from many single cells to identify determinants of cell-to-cell chromatin variation [54]. As the ends of each fragment are indicative of regions of open chromatin, it is possible to analyze the combined signal from these fragments to determine regions of the genome enriched for open chromatin and thus, putatively of regulatory and functional significance. However, a theoretical disadvantage of such a method would be not being able to identify rare peaks appearing only in scarce populations. One major limitation to current scATAC-seq approaches is that they capture only a subset of the open chromatin sites in single cells, a lot of sites may be lost or not detectable during both experimental and computational procedures. It seems unlikely that more comprehensive coverage can be achieved in the near future. Higher per-cell coverage would allow new questions to be answered. For example, it is still confusing how chromatin accessibility differs between the two alleles in a single cell, or how do open chromatin regions correlate in an individual cell [95].

### Prospects for spatial epigenomes

Tn5 transposase is widely used for chromatin accessibility sequencing, linear amplification via transposon insertion [96] and even detection of potential pathogens co-infected with coronavirus [97]. The ability of Tn5 to anneal a variety of adapters for personalized applications is an essential aspect of proposing innovative research designs, such as coupling adapters with fluorescence for accessible chromatin imaging [84]. Besides, modifications to Tn5 and the design of protocols would further realize high-throughput and large-scale experiments as undisclosed reagents severely limit the development of novel applications [98]. If scientists could efficiently personalize library preparation procedures, it would allow epigenome sequencing technology to benefit more large-scale projects like The Encyclopedia of DNA Elements (ENCODE) [99, 100]. For example, draw upon the principles of the spatial transcriptome [101], once Tn5 is available in bulk, it may be possible to sequence samples directly without cell dissociation and centrifugation steps, thereby retaining the original state of cells and significantly reducing loss. Also, spatial epigenomes can be realized by mapping the chromatin states of individual cells to their location information. Although many studies have yielded an excellent temporal map of tissue and organ development, the challenge remains to improve further the spatial resolution of developmental studies, such as cell migration trajectories in embryonic organs at different stages.

Not only the spatial location and migration of cells are required, but also the spatial dynamics on the molecular level is crucial. Genome-wide chromatin conformational changes within cells are important mechanisms for regulating cell behavior [102]. The HiChIP technology has successfully resolved the dynamics of chromatin structure with high sensitivity and high resolution by combining ChIP with chromatin conformation capture [103]. Therefore, with the further development and improvement of the technologies involved in analyzing the dynamics of the epigenome, there are broad and attractive prospects to integrate different omics and experimental techniques for multi-dimensional life science research. A more comprehensive map would also provide a better understanding of the interplay of multiple regulatory elements within individual cells.

## Data Availability

Not applicable.

## Abbreviations

ATAC-see: Assay of transposase-accessible chromatin with visualization
ATAC-seq: Assay for transposase-accessible chromatin with high-throughput sequencing
ChIP: Chromatin immunoprecipitation
ChIP-chip: Chromatin immunoprecipitation (ChIP) followed by microarray hybridization (chip)
ChIP-seq: Chromatin immunoprecipitation sequencing
CoBatch: Combinatorial barcoding and targeted chromatin release
CUT&RUN: Cleavage under targets and release using nuclease
CUT&Tag: Cleavage under targets and tagmentation
DNase-seq: DNase I hypersensitive site sequencing
Drop-ChIP: Droplet-based single-cell ChIP-seq
ecDNA: Extrachromosomal DNA
FACS: Fluorescence activated cell sorting
GEM: Gel bead in emulsion
IFC: Integrated fluidic circuit
MNase: Micrococcal nuclease
MNase-seq: MNase digestion with deep sequencing
pA/G-Tn5: Protein A/G-fused Tn5 transposase
RNA-seq: RNA sequencing
scATAC-seq: Single-cell ATAC-seq
scChIP-seq: Single-cell ChIP-seq
sci-ATAC-seq: Single-cell combinatorial indexing assay for transposase accessible chromatin with sequencing
sci-CAR: Single-cell combinatorial indexing-based co-assay of chromatin accessibility and mRNA
sc-itChIP-seq: Single-cell simultaneous indexing and tagmentation-based ChIP-seq
SNARE-seq: Single-nucleus chromatin accessibility and mRNA expression sequencing
ZGA: Zygotic genome activation
